# Sonophotocatalytic dimerization of coniferyl alcohol – The influence of energy distribution during sonication^☆^

**DOI:** 10.1016/j.ultsonch.2026.108020

**Published:** 2026-08-22

**Authors:** Marta Paszkiewicz-Gawron, Pablo Martinez-Marco, Dariusz Łomot, Beata Naumczuk, Behdokht Hashemi Hosseini, Juan Carlos Colmenares

**Affiliations:** a Institute of Physical Chemistry, Polish Academy of Science, Kasprzaka 44/52, 01-223 Warsaw, Poland; b Institute of Organic Chemistry, Polish Academy of Science, Kasprzaka 44/52, 01-223 Warsaw, Poland

**Keywords:** Acoustic field, Sonophotocatalysis, Coniferyl alcohol, Dimerization, Organic synthesis, DHCA

## Abstract

This communication presents a novel and sustainable approach to organic synthesis based on the sonophotocatalytic dimerization of coniferyl alcohol. The sonophotocatalytic approach synergistically integrates ultrasonic cavitation with light-induced activation, significantly enhancing reaction efficiency and selectivity compared to conventional multi-step procedures. Under optimized conditions, a chitosan-lignin-based hybrid catalyst and/or a ZnO catalyst functionalized with Cu achieved 100% conversion of coniferyl alcohol with high selectivity toward the dimeric product racemic dehydrodiconiferyl alcohol (DHCA), demonstrating remarkable stability over a 24-hour time-on-stream. For the first time, coniferyl alcohol dimers were successfully synthesized using low-powered ultrasound (at ambient temperature and pressure) instead of the harsher conditions typically required in traditional dimerization reactions, such as high temperatures, elevated pressures, and the use of oxidizing agents or toxic catalysts. The correlations between acoustic fields and catalytic properties were studied, and described. Consequently, it was demonstrated that selectivity can be controlled by modulating the sonication energy input. The dimeric products obtained possess considerable value for pharmaceutical and chemical applications. This methodology represents a paradigm shift toward sustainable organic synthesis, valorizing lignocellulosic waste, as well as the production of valuable chemicals through environmentally benign catalytic processes. The approach aligns with circular economy principles and offers an alternative for the synthesis for coniferyl alcohol dimers using conventional multi-step synthesis.

## Introduction

This study presents the sonophotocatalytic dimerization of coniferyl alcohol, using chitosan-lignin-based hybrid catalytic complex functionalized with ZnO and copper. The approach described not only reduces the current dependence on fossil-based inputs, but it also valorizes lignocellulosic byproducts, thereby promoting the circular economy [1]. Furthermore, the system exhibits significant improvements in catalyst performance, selectivity, and stability when compared to traditional methods. The dimeric products obtained constitute high-value compounds for industrial and pharmaceutical applications, demonstrating the potential of this methodology as a clean and potentially scalable synthetic route. Conventional dimerization methods are often multi-step procedures which require harsh conditions, including high temperatures, elevated pressures, and the use of oxidizing agents or toxic catalysts, such as pyridine, acetic anhydride, triethyl phosphoacetate, cyclohexane, peroxidase, FeCl_3_, Et_3_N and methanol. This results in high energy costs and serious environmental concerns, thereby limiting their adoption in more sustainable industrial processes [2], [3], [4], [5]. To date, only one publication has been concerned with the photochemical polymerization of coniferyl alcohol. However, in this case, the authors used a 120 W low-pressure mercury lamp as the UV irradiation source, which is environmentally unfavorable due to its high energy consumption and reliance on toxic mercury [6]. In the present study, we utilized an LED lamp as the irradiation source, which is environmentally favorable due to its low energy consumption and the absence of mercury [7], [8]. Existing catalytic systems for coniferyl alcohol dimerization rely on metal- or organocatalyzed oxidative radical generation, and are based on transition metal catalysts, palladium-based catalysts, organocatalytic catalysts (nitroxyl radicals) and hypervalent iodine oxidants. They remain limited, however, by poor selectivity in the subsequent coupling step, underscoring the need for more controlled strategies [9], [10], [11].

The interaction between acoustic cavitation and the light-induced excitation of semiconductor materials promotes the efficient generation of reactive species and improves the catalyst and light dispersion. Mechanistically, sonophotocatalysis facilitates the controlled activation of the substrate and the formation of key radicals for dimerization, reducing the need for extreme conditions and resulting in minimal waste generation [12], [13], [14], [15]. Recently, ultrasound has been increasingly employed for the synthesis of bioactive natural products [16], [17].

**Fig. 1:**
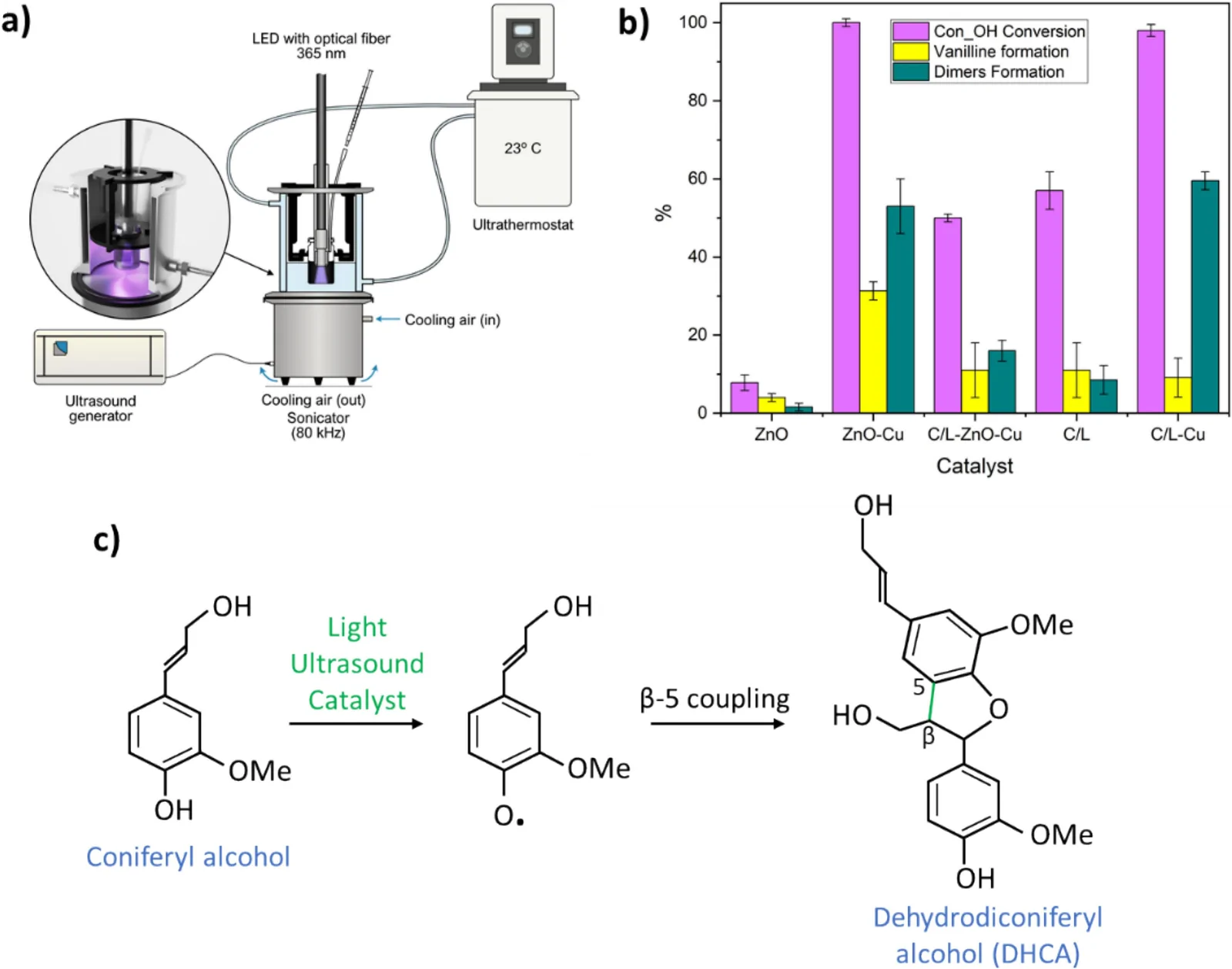
a) Schematic representation of the sono-photo reactor system with an ultrasound generator, optical fiber light and thermostat), b) Coniferyl alcohol conversion to dimers and vanillin after 180 min of reaction using 80 kHz sonicator with 20% amplitude, c) Scheme for the formation of a dimeric product – DHCA from coniferyl alcohol monomer.

Under optimized conditions (Fig. 1a), a SinapTec sonicator, with a frequency of 80 kHz, 20% amplitude was used, with pulse settings (10 s work/5 s off), a LEDMOD V2 Omicron-Laserage lamp, λ = 365 nm with an optical fiber with an irradiance of 0.197 W/cm^2^, 3 h, 23 °C with an ultrathermostat, cooling air for the electronic part of the sonicator. The reaction involved 15 mL of 1 mM coniferyl alcohol solution in acetonitrile, with 15 mg of each catalyst. Samples were collected at 0, 30, 60, 120, and 180 min. The vial in which the reactions were conducted was placed 15 mm above the transducer. This was considered necessary as at this distance one of the acoustic pressure maxima occurs for the wavelength we applied (Fig. S1). The samples were analyzed using an Ultra-Performance ACQUITY UPLC I-Class (*Waters Inc*), coupled with a Synapt G2-S HDMS (*Waters Inc*) mass spectrometer, equipped with an electrospray ion source and a q-TOF type mass analyzer (details are included in the Supplementary Data section) to identify the main reaction products. ZnO-Cu and C/L-Cu catalysts achieved 100% and 98% conversion of coniferyl alcohol, with a high selectivity toward dimeric product – dehydrodiconiferyl alcohol (DHCA), 54% and 59% (*m*/*z* = 357), respectively, and vanillin 31% and 9%, respectively (*m*/*z* = 151) (Fig. 1b). Each catalyst was tested three times for coniferyl alcohol dimerization. Error bars (based on three replicates) for the conversion of coniferyl alcohol, as well as DHCA and vanillin formations, are presented in Fig. 1b.

The coniferyl alcohol dimerization product – racemic DHCA − obtained can be used as a wound-healing aid [18]. It has been found that DHCA can promote the healing of scalp wounds in mice by enhancing epithelial cell proliferation and collagen formation and reducing inflammatory cell infiltration [18]. In further studies, DHCA was developed as an efficient therapeutic for osteoporosis by regulating osteoblastogenesis through its estrogenic effects. Dehydrodiconiferyl alcohol has also been shown to bind to an estrogen receptor, and indeed it exhibits several of the activities of estrogen, such as its anti-inflammatory and anti-oxidative stress effects [19]. Globally, vanillin is one of the most widely used flavor compounds, appearing in food, cosmetics and agrochemicals, as well as in pharmaceuticals, where it is used as a flavoring agent to improve the palatability of oral medications. Vanillin also serves as a substrate for synthesizing fine chemicals and aromatic compounds [20] in several other industries.

In general, coniferyl alcohol dimers can exist in three isomeric forms, which can be described by their coupling type: β-5, otherwise known as dehydrodiconiferyl alcohol, β-O-4, otherwise known as guaiacylglycerol-coniferyl alcohol ether, and the β-β form, known as pinoresinol [21]. Our experiments show that changing the acoustic energy input during sonication results in precise control over product distribution and selectivity in the sonophotocatalytic dimerization of coniferyl alcohol. This is achieved by altering the cavitation dynamics and catalyst activation pathways. Using an 80 kHz ultrasound frequency set at a 30% amplitude, we achieved a 100% conversion of coniferyl alcohol, obtaining 14 distinct products, including dimers – DHCA (*m*/*z* = 357), trimers (*m*/*z* = 537) of coniferyl alcohol, and vanillin (*m*/*z* = 151), along with several other products such as ferulic acid (*m*/*z* = 193), coniferyl aldehyde (*m*/*z* = 177) and 3,4-dihydroxybenzylaldehyde (*m*/*z* = 137) (detailed chromatograms are shown in Fig. S2). When the amplitude was reduced from 30% to 20%, with a corresponding reduction in the energy input from an estimated power intensity of 13.9 ± 0.3 W to 13.4 ± 0.3 W (estimated power values were calculated based on pressure divided by impedance of the medium – detailed information is included in the Supplementary Data – Figure S3 Tables S1–S7), a 100% conversion was still achieved; however, only 5 products were obtained, where the main products were DHCA (54% selectivity) and vanillin (31% selectivity) –see the Table1. Importantly, no trimers were formed at an amplitude of 20%. A further decrease in the sonication amplitude to 10% resulted in a reduced conversion of 93%, yielding 13 different products overall. In this case, an additional dimeric isomer, pinoresinol (having the same *m*/*z* = 357 as DHCA but a different retention time), was also observed. Based on these findings, an amplitude of 20% was selected as preferable to 30% or 10%. Notably, the overall conversion of coniferyl alcohol was higher at 20% amplitude (100%) than at 10% amplitude (93%).

**Table 1:** The optimization results for the selection of amplitude at the frequency of 80 kHz of sonication using ZnO-Cu sample at ambient temperature and pressure.

Amplitude at the frequency of 80 kHz	Conversion of coniferyl alcohol monomers (%)	Selectivity (%)		Total number of the reaction products	Trimers
		DHCA	Vanillin		
10%	93 ± 2	59 ± 4	25 ± 3	13	**+**
20%	100 ± 1	54 ± 5	31 ± 4	5	**−**
30%	100 ± 1	52 ± 5	20 ± 4	14	**+**

Our experimental results strongly suggest that the dimerization of coniferyl alcohol to its corresponding dimers (including β–β, β–O–4, and β–5 structures) proceeds via a radical mechanism. Scavenger experiments were performed using the ZnO/Cu catalyst with different types of scavengers (80 kHz and 20% amplitude, using LEDMOD V2 Omicron-Laserage lamp λ = 365 nm with optical fiber with irradiance 0.197 W/cm^2^, in each experiment we used a 1 mM concentration of coniferyl alcohol with a 1 mM concentration of a specific scavenger): *tert*-butyl alcohol (hydroxyl radical scavenger •OH), AgNO3 (electron scavenger), p-benzoquinone (superoxide anion radical scavenger O2^•−^), diammonium oxalate monohydrate (hole scavenger), TEMPO (2,2,6,6-tetramethylpiperidine-1-oxyl) – a reagent that quenches many types of radicals [22]. Based on the obtained results, we confirmed that the dimerization mechanism occurs via radicals, but it was not entirely clear which reactive species were responsible. The significant inhibition of the reaction by both TEMPO and p-benzoquinone suggests that radical species, particularly superoxide radicals (O_2_•^−^), play a crucial role in the mechanism. However, further studies, such as EPR spectroscopy, would be required to definitively confirm the specific radical intermediates. These species can arise from atmospheric oxygen and typically induce mild oxidative conditions, in contrast to the highly reactive hydroxyl radicals **·**OH. Consequently, O_2_^•−^ promotes radical coupling and dimer formation rather than extensive oxidative degradation of the coniferyl alcohol molecule. It is noteworthy that TEMPO can quench many types of radicals, including the carbon-centered radicals R**·**, but it is less reactive towards oxy radicals, e.g. hydroxyl radicals **·**OH [23], [24]. TEMPO forms a strong bond with other types of radicals. The observed near-complete suppression of coniferyl alcohol conversion in the presence of TEMPO appears to confirm that radical intermediates are crucial in the reaction mechanism, as TEMPO effectively blocks radical formation and thereby inhibits substrate conversion (Figure S4). As scavenger studies can be misleading, we verified the radical mechanism using the additional mass spectrometry experiment described below.

**Fig. 2:**
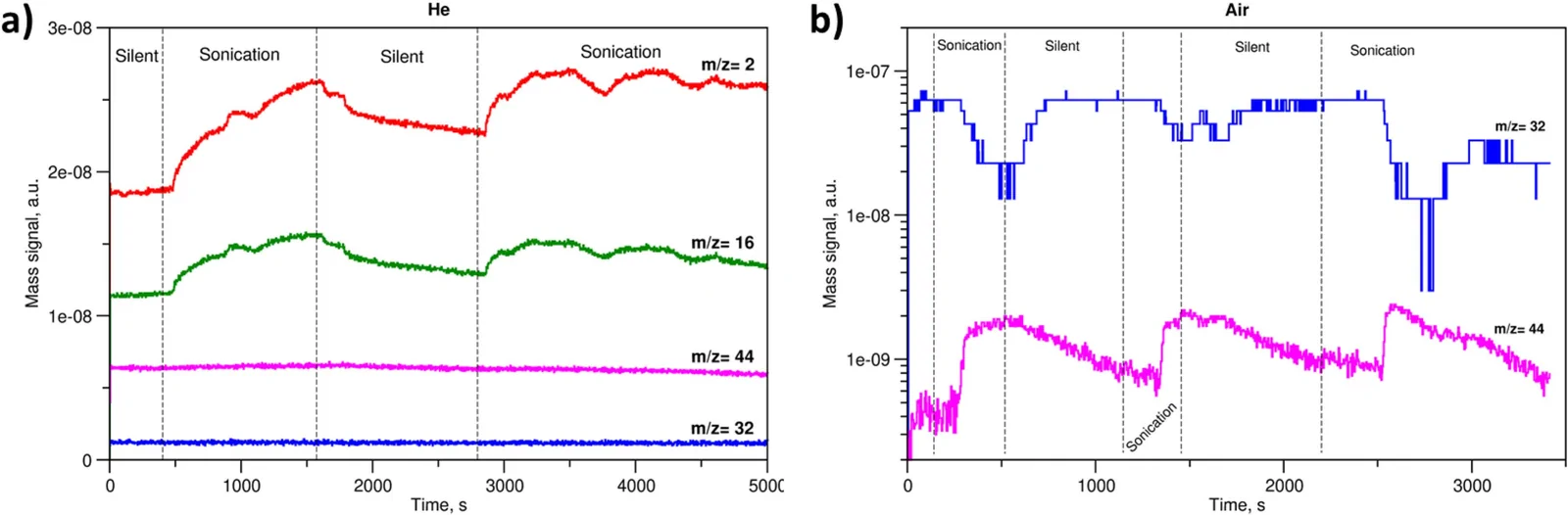
Sonolysis of acetonitrile and radical formation under a) inert conditions (He flow = 25 ml/min), b) oxidative conditions (Air flow = 25 ml/min).

To investigate whether sonication induces radical generation from pure acetonitrile, the reaction system was coupled to an online mass spectrometer. Mass spectrometric analysis revealed molecular fragmentation of acetonitrile under ultrasound, indicating the formation of reactive radical species. In particular, the detection of methane (*m*/*z* = 16) under inert conditions (He flow = 25 ml/min), which is consistent with the rupture of the C-C bond, suggests the generation of methyl radicals (•CH_3_), which may also contribute to the observed dimerization process (Fig. 2a). The production of hydrogen *m*/*z* = 2 suggests an efficient cleavage of the C-H bonds. Importantly, both methane and hydrogen were generated once sonication was used. Under silent conditions (absence of sonication), the concentrations of both gases decreased. Under inert conditions, the concentrations of oxygen *m*/*z* = 32 and carbon dioxide *m*/*z* = 44 remained at zero. However, under oxidizing conditions (synthetic air flow = 25 ml/min), carbon dioxide levels increased in response to ultrasound, while oxygen levels decreased as it was consumed (Fig. 2b). When sonication was halted, the concentration of carbon dioxide gradually decreased, eventually returning to its initial value. In contrast, no signals attributable to •CN radicals were detected. This observation is likely related to the high dissociation energy of the C≡N bond in acetonitrile, which may not be efficiently cleaved under the applied sonication conditions (80 kHz). Importantly, similar findings regarding the sonolysis of pure organic liquids were published by Weissler et al. in 1965 [25], [26]. Please see the Fig. 2.

Overall, these results demonstrate that sonication of acetonitrile leads to the formation of radical species. However, the generated radicals appear to be highly transient and insufficiently stable to promote the dimerization reaction in the absence of a catalyst. Preliminary control experiments performed using only acetonitrile and coniferyl alcohol, without the catalyst, showed no detectable conversion of the monomer (Figure S6). These findings suggest that the catalyst plays a crucial role in stabilizing and/or activating the sonochemically generated radical intermediates, thereby enabling their participation in the dimerization process.

**Fig. 3:**
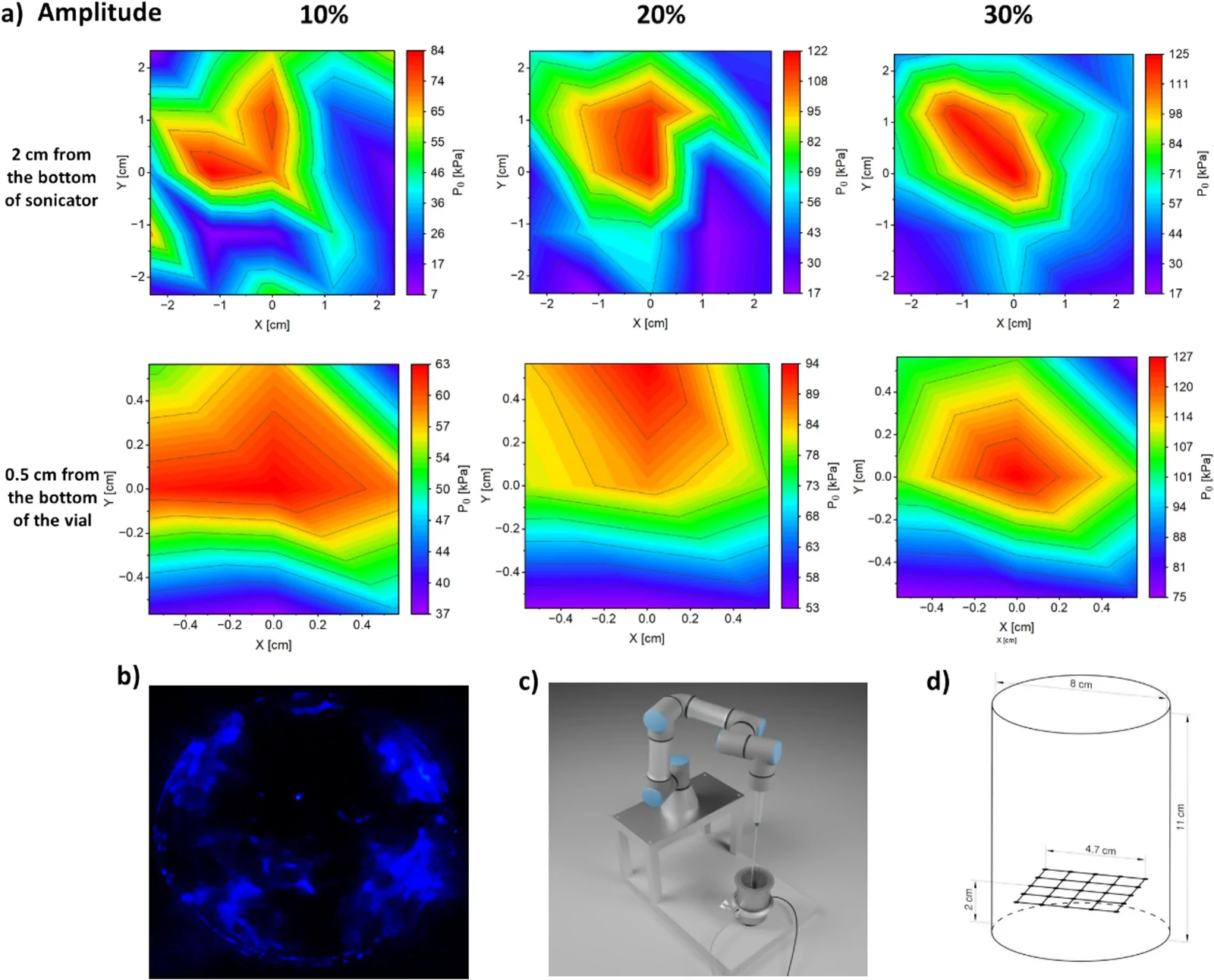
a) Mapping of direct pressure p_0_, 2 cm and 0.5 cm from the bottom of the sonicator with the application of different amplitudes of sonication: 10, 20 and 30%. On the X and Y axes, the distances are shown from the center of the sonicator or vial. The mapping of direct pressure P_0_, 0.5 cm from the bottom of the vial; b) A photograph of the effect of input power in the sonicator using luminol solution, c) Robotic arm – the device employed for performing the mapping of energy distribution, d) Schematic view of the sonicator SinapTec with different locations for measurements of acoustic energy.

An essential consideration in sonochemistry is accurately determining the amount of energy delivered to the system. This step is challenging and very rarely found in experimental publications related to the use of ultrasound. Therefore, the initial task involved mapping the ultrasound field to verify that the waves were effectively penetrating the reaction vessel (Fig. 3a). The distribution of ultrasound acoustic fields were done with the use of a hydrophone.

The higher selectivity of coniferyl alcohol dimerization at an amplitude of 20% can be better understood by considering the interplay between the acoustic pressure distribution, cavitation intensity and the radical-mediated reaction pathway. Direct pressure mapping was performed using an HCT-0320 hydrophone and an MCT-2000 cavitation meter, assisted by a robotic arm to localize points in 3D space. The internal diameter of the reactor was 8 cm and length 11 cm. The transducer is located at the bottom of the reactor. For measurement of direct pressure, the volume of sonicator with water (150 ml) was divided by five planes in all three spatial directions at distances of 0.5, 1.0, 1.5, 2.0, and 2.5 cm from the bottom. Averaging time for every point of measurement was 5 s. In the case of the vial with diameter 2 cm and length 5 cm, the volume with water (15 ml) was divided by three in each 3D planes at distances of 0.5, 1.0, and 1.5 cm from the bottom. Averaging time for every point of measurement was the same as above. For simplification, we presented collected data from planes above 2 and 0.5 cm from bottom of the sonicator and vial, respectively. Schematic representation of sonicator SinapTec with different locations for acoustic energy, is shown in Fig. 3d.

Acoustic field maps show that at an amplitude of 10%, the sonication energy is insufficiently intense and highly spatially irregular, with the maximum direct pressure of 63 kPa at a position of 0.5 cm from the bottom of the vial (Fig. 3a). The pressure field in this setting is dominated by localized hot spots, surrounded by regions of much weaker sonication at a position of 2 cm from the bottom of the sonicator, with a maximum of 84 kPa. These conditions generate infrequent and unevenly distributed cavitation events. The specific distribution of direct pressure likely leads to inefficient radical formation. Consequently, the radical concentration is expected to be low and undergoes strong spatial fluctuations and competing side reactions – such as disproportionation or polymerization initiated by uncontrolled radical encounters. Supporting this hypothesis, coniferyl alcohol trimers were detected by UPLC-MS (*m*/*z* = 537) when an amplitude of 10% was applied (Table 1). Additionally, an additional dimeric isomer − pinoresinol (the same *m*/*z* = 357 as DHCA but a different retention time) was observed.

In contrast, at 30% amplitude the acoustic environment becomes excessively energetic, with a maximum direct pressure of 127 kPa both in the center of the vial and in the center of the sonicator. The pressure field becomes heavily concentrated, particularly at short distances from the sonicator tip, where the hydrophone measurements indicate a nearly homogeneous high–intensity sonication. Such high energy densities promote extremely vigorous cavitation, increasing the probability of bubble collapse events and the creation of transient microenvironments with significantly elevated temperatures, pressures and shear forces. Under these conditions, radical concentrations likely become excessively high, consequently shortening the lifetime of reactive species to maintain selective coupling. The reaction medium likely becomes dominated by uncontrolled radical cascades and chain reactions. High cavitation intensity also enhances local turbulence and mechanical disruption, which increases the likelihood of secondary and tertiary reactions, including over–oxidation, oligomerization, and the formation of multiple competing dimeric and trimeric products. This explains why the selectivity deteriorates when the amplitude is raised to 30%. Further evidence for this hypothesis was provided by the detection of coniferyl alcohol trimers at an applied amplitude of 30% by UPLC-MS (Table1).

The optimal selectivity observed at an amplitude of 20% can be attributed to an intermediate acoustic regime, which corresponds to ∼78 kPa in the center of the vial; this probably balances the competing factors of radical generation efficiency and reaction control. The pressure field at this amplitude is significantly more uniform and centrally organized than at 10%, reflecting the excessive intensity observed at 30%. This acoustic environment produces cavitation at an intensity sufficient to generate coniferyl alcohol radicals at a controlled rate, while avoiding the destructive or highly chaotic cavitation associated with higher amplitudes. In this regime, the radical concentration probably remains within a window that favors the specific coupling pathway leading to the observed dimer, and the radicals persist long enough to undergo selective recombination rather than uncontrolled propagation or excessive oxidation. The spatial coherence of the acoustic field at 20% likely ensures a more uniform production of reactive molecules, which further supports selective pathways by reducing the formation of localized areas of extremely high radical density.

Together, these observations suggest that an amplitude of 20% corresponds to an optimal selectivity level whenever cavitation activity is intense enough to initiate the desired radical processes, but not so intense as to induce any excessive side reactions. This amplitude creates a well-defined acoustic pressure field, which possesses sufficient energy to promote efficient dimerization while maintaining a level of control that suppresses nonselective radical pathways (e.g. the formation of trimers).

An additional experiment that we performed used 1 g/L of luminol (3-aminophthalhydrazine) water solution. This allowed us to visualize the locations at which ·OH radicals are generated within the sonoreactor (Fig. 3b). Oxidizing species such as ·OH radicals generated by cavitation react with luminol and emit visible light (λ = 430 nm, blue light). The images were taken with a digital camera under ultrasound irradiation in a completely dark room; the exposure time was 30 s. The experiments related to determining the relative pressure were conducted using the hydrophones positioned with a robotic arm – specialist equipment for mapping of energy distribution – Fig. 3c.

To sum up, under optimized sonophotocatalytic conditions (80 kHz frequency 20% amplitude, which corresponds to an estimated intensity of 13.4 ± 0.3 W, 365 nm with LED irradiance 0.197 W/cm^2^), coniferyl alcohol was efficiently converted with a 100% conversion rate and a 54% selectivity toward the dimeric product – DHCA and 31% of vanillin, using ZnO–Cu and C/L–Cu catalysts, demonstrating remarkable stability over time-on-stream of 24 h (Fig. S7). The combined action of acoustic cavitation and the photoexcitation of semiconductor materials enhanced the formation of reactive species and improved both the catalyst and light dispersion. More importantly, light irradiation alone was found to produce active species such as radicals, and the introduction of ultrasound further amplified this process. Mechanistically, sonophotocatalysis enables more controlled substrate activation and promotes the generation of the key radical intermediates required for dimerization, thereby reducing the need for harsher reaction conditions and resulting in minimal waste formation.

Product distribution was strongly dependent on the ultrasonic amplitude, demonstrating that the modulation of the acoustic energy input enables precise control over the reaction selectivity. Higher amplitudes promoted the formation of multiple products, including trimers, whereas lowering the power intensity from 13.9 ± 0.3 W to 13.4 ± 0.3 W (20% amplitude) resulted in complete conversion, with the selective formation of dimers and vanillin, along with the suppression of trimer formation. Mechanistic studies, including on–line mass spectrometry, radical scavenging experiments with TEMPO, and acoustic field mapping, provide compelling evidence for radical mechanism of dimerization. These findings highlight the critical role of controlled sonication in directing product selectivity in the radical dimerization of coniferyl alcohol, and serves as green alternative to the conventional multi-step synthesis of coniferyl alcohol dimers.

## CRediT authorship contribution statement

**Marta Paszkiewicz-Gawron:** Writing – original draft, Investigation, Data curation, Conceptualization. **Pablo Martinez-Marco:** Visualization, Investigation, Data curation. **Dariusz Łomot:** Visualization, Methodology, Investigation, Data curation. **Beata Naumczuk:** Methodology, Investigation. **Behdokht Hashemi Hosseini:** Visualization, Investigation. **Juan Carlos Colmenares:** Writing – review & editing, Project administration, Conceptualization.

## Declaration of competing interest

The authors declare that there are no known competing interests or personal relationships that could have appeared to influence the research work reported in this manuscript.
