# Supplementary material for: Sonophotocatalytic dimerization of coniferyl alcohol – The influence of energy distribution during sonication
Source: Ultrason Sonochem. 2026 Aug 22;133:108020. doi: 10.1016/j.ultsonch.2026.108020 (PMC13524724; doi:10.1016/j.ultsonch.2026.108020)
Supplement: Supplementary file 1 [file mmc1.docx]

Supplementary Data

COMMUNICATION

Sonophotocatalytic dimerization of coniferyl alcohol – the influence of energy distribution during sonication

Marta Paszkiewicz-Gawron ^1^, Pablo Martinez-Marco ^1^, Dariusz Łomot ^1^, Beata Naumczuk ^2,^ , Behdokht Hashemi Hosseini ^1^, Juan Carlos Colmenares ^1*^

^1^ Institute of Physical Chemistry, Polish Academy of Science, Kasprzaka 44/52, 01-223 Warsaw, Poland

^2^ Institute of Organic Chemistry, Polish Academy of Science, Kasprzaka 44/52, 01-223 Warsaw, Poland

***** Correspondence: [jcarloscolmenares@ichf.edu.pl](mailto:jcarloscolmenares@ichf.edu.pl), mpaszkiewicz-gawron@ichf.edu.pl

## **1 Materials**

Zinc acetate dihydrate (Zn(CH₃COO)₂·2H₂O, Sigma-Aldrich, ≥99%) CAS No. 5970-45-6, copper acetate monohydrate (Cu(CH₃COO)₂·H₂O, ABCR Lab, 98%) CAS No. 6046-93-1, sodium hydroxide (NaOH, StanLab, ≥99%) CAS No. 1310-73-2, chitosan (medium MW, Sigma-Aldrich) CAS No. 9012-76-4, alkali lignin (Sigma-Aldrich, low sulphonate content) CAS No. 8068-05-1 and acetic acid (CH₃COOH, Chempur, 99.5%) CAS No. 64-19-7 were used as received. MQ-water (18.2 MΩ cm).

## **1.2 Catalyst Preparation**

ZnO: Zinc acetate dihydrate (0.20 mM) was dissolved in 150 mL deionized water. NaOH (0.15 M) was added dropwise under stirring until a white precipitate formed. The solid was immediately collected by centrifugation (6000 rpm, 10 min), washed with deionized water, oven-dried at 110 °C for 12 h, and calcined in air at 400 °C (2 °C min⁻¹) for 4 h.

ZnO–Cu: The ZnO-Cu catalyst was prepared by a co-precipitation method adapted from the procedure reported by Wang et al. [1] For ZnO-Cu catalysis. Aqueous solutions containing 0.1 mM Zn(CH3COO)2•H2O and 0.05 mM Cu(CH3COO)2•H2O were used as metal precursors. These solutions were simultaneously added to deionized water under vigorous stirring, followed by dropwise addition of 0.15M NaOH as the precipitant. The resulting slurry was aged at 80°C for 2h. The precipitate was then filtered, thoroughly washed with deionized water, and dried at 110°C for 12h.

C/L (Chitosan-Lignin): Chitosan-lignin (C/L) composites were prepared by a hydrothermal method adapted from Khan et al. [2] Alkali lignin (130 mg) and chitosan (1.20 g) were dispersed in 1 wt% aqueous acetic acid solution and sonicated at 100 kHz for 30 min to form a homogeneous suspension.

The suspension was transferred to a Teflon-lined stainless steel autoclave and subjected to hydrothermal treatment at 180 °C for 12 h. After natural cooling to room temperature, the obtained blackish-brown solid was filtered, washed thoroughly with deionized water and dried at 110 °C for 12 h.

C/L-Cu: C/L-Cu composites were prepared analogously to C/L samples, with the addition of Cu(CH₃COO)₂·H₂O (0.05 mM) to the chitosan–lignin suspension in 1 wt% acetic acid prior to sonication and hydrothermal treatment.

After dispersing alkali lignin (130 mg) and chitosan (1.20 g) in 1 wt% aqueous acetic acid, Cu(CH₃COO)₂·H₂O (0.05 mM) was introduced to the suspension, followed by sonication at 100 kHz for 30 min. The resulting mixture was transferred to a Teflon-lined stainless steel autoclave and subjected to hydrothermal treatment at 180 °C for 12 h. The obtained solid was filtered, washed thoroughly with deionized water, and dried at 110 °C for 12 h.

C/L–ZnO–Cu: The hybrid catalyst C/L–ZnO–Cu was synthesized by combining the co-precipitation procedure of ZnO–Cu with the hydrothermal method of C/L composites [1], [2].

Pre-synthesized C/L composite was dispersed in water together with Zn(CH₃COO)₂·2H₂O (0.10 mM) and Cu(CH₃COO)₂·H₂O (0.05 mM), followed by sonication at 100 kHz for 45 min to ensure homogeneous metal incorporation. The suspension was transferred to a Teflon-lined stainless steel autoclave and subjected to hydrothermal treatment at 180 °C for 12 h. The resulting solid was filtered, thoroughly washed with deionized water, and dried at 110 °C for 12 h.

[1] Wang, Z.-j.; Song, H.; Pang, H.; Ning, Y.; Dao, T. D.; Wang, Z.; Chen, H.; Weng, Y.; Fu, Q.; Nagao, T.; Fang, Y.; Ye, J. Photo-assisted methanol synthesis via CO₂ reduction under ambient pressure over plasmonic CuZnO catalysts. Appl. Catal. B: Environ. 2019, 250, 10–16. <https://doi.org/10.1016/j.apcatb.2019.03.003>

[2] Khan A, Goepel M, Lisowski W, Żółtowski D, Lisovytskiy D, Mazurkiewicz-Pawlicka M, et al. Titania-chitosan-lignin nanocomposite as an efficient photocatalyst for the selective oxidation of benzyl alcohol under UV and visible light. RSC Adv. 2021;11:34996-500.

1.3 UPLC-MS Analysis

The UPLC-MS analyses were performed using Ultra-Performance Liquid Chromatograph ACQUITY UPLC I-Class (*Waters Inc*) coupled with Synapt G2-S HDMS (*Waters Inc*) mass spectrometer equipped with an electrospray ion source and q-TOF type mass analyzer.

Chromatographic separations were carried out using the Acquity UPLC BEH C18 column (2.1 × 100 mm, 1.7 µm, *Waters*). Gradient elution was started from 5% B to 100 % B in 12 min, then 100% B kept constant for 2 minutes, and finally return to the initial conditions, at a ﬂow rate of 0.3 mL/min. Eluent A was 10 mM ammonium acetate and eluent B was acetonitrile. The UV chromatograms were recorded at 260 nm and 365 nm.

The High Resolution spectra in negative mode were performed with capillary voltage set to 2.0 kV. The desolvation gas flow was 600 L/h and temperature 350 °C. The sampling cone voltage was 40V and source offset was set to 40 V and the source temperature was 120 °C.

The data were obtained in a scan mode ranging from 100 to 1200 m/z.

The Leucine-Enkephaline solution was used as the Lock-Spray reference material.

The instrument was controlled and recorded data were processed using *MassLynx V4.2* software package (*Waters Inc*).

**Figure S1 Scheme of the sonication system with dimensions.**

**Figure S2 The results of LC-MS analysis.**

**Figure S3 Schematic view of the sonicator SinapTec and different locations for measurements**.

Mapping was performed using hydrophone HCT-0320 and cavitation meter MCT-2000 with help of a robotic arm to localize points in 3D space.

The internal diameter of the reactor was 8 cm and length 11 cm. Transducer is located at the bottom of the reactor. For measurement of direct pressure, the volume of sonicator with water (150 ml) was divided by five planes in each 3D directions. With the distance from the bottom 0.5, 1.0, 1.5, 2.0 and 2.5 cm. Averaging time for every point of measurement was 5 s.

In case of vial with diameter 2 cm and length 5 cm, the volume with water (15 ml) was divided by three in each 3D planes. With the distance from the bottom 0.5, 1.0 and 1.5. Averaging time for every point of measurement was the same as above. For simplification, we presented collected data from planes above 2 and 0.5 cm from bottom of the sonicator and vial, respectively.

**Table S1 The nominal and estimated power [W] under different amplitudes.**

| SinapTec 80 kHz | | |
| --- | --- | --- |
| Amplitude [%] | Nominal power [W] | Estimated power [W] |
| 10 | 5 | 6.9 ±0.1 |
| 20 | 10 | 13.4 ±0.3 |
| 30 | 15 | 13.9 ±0.3 |

Estimated values are calculated on the base of pressure divided by impedance of the medium, but without valid assumption as big distance between the source and the sensor and lack of reflections.

**Table S2 Data for estimated power calculations with 10% amplitude.**

| **A 10%** | | | |
| --- | --- | --- | --- |
| **X** | **Y** | **P0 [kPa]** | **I [W/cm^2^]** |
| -2.33 | -2.33 | 12.9 | 0.0111 |
| -1.17 | -2.33 | 24.8 | 0.0412 |
| 0.00 | -2.33 | 53.3 | 0.1903 |
| 1.17 | -2.33 | 34.8 | 0.0811 |
| 2.33 | -2.33 | 20 | 0.0268 |
| -2.33 | -1.17 | 66.3 | 0.2945 |
| -1.17 | -1.17 | 9.1 | 0.0055 |
| 0.00 | -1.17 | 12.2 | 0.0100 |
| 1.17 | -1.17 | 45.1 | 0.1363 |
| 2.33 | -1.17 | 17.4 | 0.0203 |
| -2.33 | 0.00 | 32 | 0.0686 |
| -1.17 | 0.00 | 83.8 | 0.4705 |
| 0.00 | 0.00 | 72.3 | 0.3502 |
| 1.17 | 0.00 | 29.8 | 0.0595 |
| 2.33 | 0.00 | 12 | 0.0096 |
| -2.33 | 1.17 | 56.4 | 0.2131 |
| -1.17 | 1.17 | 55.1 | 0.2034 |
| 0.00 | 1.17 | 77.5 | 0.4024 |
| 1.17 | 1.17 | 24.6 | 0.0405 |
| 2.33 | 1.17 | 32 | 0.0686 |
| -2.33 | 2.33 | 7.2 | 0.0035 |
| -1.17 | 2.33 | 41.1 | 0.1132 |
| 0.00 | 2.33 | 59.3 | 0.2356 |
| 1.17 | 2.33 | 44.3 | 0.1315 |
| 2.33 | 2.33 | 60.9 | 0.2485 |

**Table S3 Calculations of estimated power for 10% amplitude.**

| **Area [cm^2^]** | **50.3** |
| --- | --- |
| **I aver. [W/cm^2^]** | **0.137** |
| **P [W]** | **6.9 ±0.1** |

**Table S4 Data for estimated power calculations with 20% amplitude.**

| **A 20%** | | | |
| --- | --- | --- | --- |
| **X** | **Y** | **P0 [kPa]** | **I [W/cm^2^]** |
| -2.33 | -2.33 | 34.9 | 0.0816 |
| -1.17 | -2.33 | 16.5 | 0.0182 |
| 0.00 | -2.33 | 56.8 | 0.2162 |
| 1.17 | -2.33 | 20.1 | 0.0271 |
| 2.33 | -2.33 | 34 | 0.0775 |
| -2.33 | -1.17 | 40 | 0.1072 |
| -1.17 | -1.17 | 60.9 | 0.2485 |
| 0.00 | -1.17 | 64 | 0.2744 |
| 1.17 | -1.17 | 18.2 | 0.0222 |
| 2.33 | -1.17 | 33.2 | 0.0739 |
| -2.33 | 0.00 | 31 | 0.0644 |
| -1.17 | 0.00 | 93.4 | 0.5845 |
| 0.00 | 0.00 | 121.3 | 0.9858 |
| 1.17 | 0.00 | 28.9 | 0.0560 |
| 2.33 | 0.00 | 64.5 | 0.2787 |
| -2.33 | 1.17 | 75.1 | 0.3779 |
| -1.17 | 1.17 | 99.6 | 0.6647 |
| 0.00 | 1.17 | 116.2 | 0.9047 |
| 1.17 | 1.17 | 85.3 | 0.4875 |
| 2.33 | 1.17 | 33 | 0.0730 |
| -2.33 | 2.33 | 63 | 0.2659 |
| -1.17 | 2.33 | 54.4 | 0.1983 |
| 0.00 | 2.33 | 73.8 | 0.3649 |
| 1.17 | 2.33 | 37.8 | 0.0957 |
| 2.33 | 2.33 | 40.6 | 0.1104 |

**Table S5 Calculations of estimated power for 20% amplitude.**

| **Area [cm^2^]** | **50.3** |
| --- | --- |
| **I aver. [W/cm^2^]** | **0.266** |
| **P [W]** | **13.4 ±0.3** |

**Table S6 Data for estimated power calculations with 30% amplitude.**

| **A 30%** | | | |
| --- | --- | --- | --- |
| **X** | **Y** | **P0 [kPa]** | **I [W/cm^2^]** |
| -2.33 | -2.33 | 17.3 | 0.0201 |
| -1.17 | -2.33 | 32.7 | 0.0716 |
| 0.00 | -2.33 | 60.2 | 0.2428 |
| 1.17 | -2.33 | 30.1 | 0.0607 |
| 2.33 | -2.33 | 23.6 | 0.0373 |
| -2.33 | -1.17 | 28.6 | 0.0548 |
| -1.17 | -1.17 | 42.6 | 0.1216 |
| 0.00 | -1.17 | 64.8 | 0.2813 |
| 1.17 | -1.17 | 53.2 | 0.1896 |
| 2.33 | -1.17 | 40.7 | 0.1110 |
| -2.33 | 0.00 | 64.5 | 0.2787 |
| -1.17 | 0.00 | 91.5 | 0.5609 |
| 0.00 | 0.00 | 124.3 | 1.0352 |
| 1.17 | 0.00 | 63.2 | 0.2676 |
| 2.33 | 0.00 | 44.3 | 0.1315 |
| -2.33 | 1.17 | 73.1 | 0.3580 |
| -1.17 | 1.17 | 119.8 | 0.9616 |
| 0.00 | 1.17 | 96.1 | 0.6188 |
| 1.17 | 1.17 | 75.1 | 0.3779 |
| 2.33 | 1.17 | 51.2 | 0.1756 |
| -2.33 | 2.33 | 49.5 | 0.1642 |
| -1.17 | 2.33 | 61.3 | 0.2518 |
| 0.00 | 2.33 | 59.7 | 0.2388 |
| 1.17 | 2.33 | 37.4 | 0.0937 |
| 2.33 | 2.33 | 54.3 | 0.1975 |

**Table S7 Calculations of estimated power for 30% amplitude.**

| **Area [cm^2^]** | **50.3** |
| --- | --- |
| **I aver. [W/cm^2^]** | **0.276** |
| **P [W]** | **13.9 ±0.3** |

**Figure S4 The results of experiments in the presence of different compounds as a radical scavenger and coniferyl alcohol with using ZnO-Cu catalyst.**

**Figure S5 Schematic representation of the role of TEMPO in catching coniferyl alcohol radicals. Based on ^1^**

(1)	Prakash, N.; Rajeev, R.; John, A.; Vijayan, A.; George, L.; Varghese, A. 2,2,6,6-Tetramethylpiperidinyloxyl (TEMPO) Radical Mediated Electro-Oxidation Reactions: A Review. *ChemistrySelect*. John Wiley and Sons Inc August 13, 2021, pp 7691–7710. https://doi.org/10.1002/slct.202102346.

**Figure S6 The results of preliminary tests of treating the coniferyl alcohol under sono, photo and sonophoto conditions in acetonitrile.**

**Figure S7 Stability test for a time-on-stream of 24 hours of the sonophotocatalytic reaction with using ZnO-Cu catalyst.**
